# Draft Genome Sequence of a Rare Pigmented *Mycobacterium avium* subsp. *paratuberculosis* Type C Strain

**DOI:** 10.1128/genomeA.01066-17

**Published:** 2017-10-12

**Authors:** Pedro Barbosa, Célia Leão, Anabel Usié, Ana Amaro, Ana Botelho, Carlos Pinto, João Inácio, Karen Stevenson, António Marcos Ramos

**Affiliations:** a Centro de Biotecnologia Agrícola e Agroalimentar do Alentejo (CEBAL)/Instituto Politécnico de Beja (IPBeja), Beja, Portugal; b Instituto de Ciências Agrárias e Ambientais Mediterrânicas (ICAAM), Universidade de Évora, Évora, Portugal; c INIAV–Instituto Nacional de Investigação Agrária e Veterinária, Lisbon, Portugal; d Moredun Research Institute, Penicuik, United Kingdom; e Serviço de Desenvolvimento Agrário de São Miguel, Ponta Delgada, Açores, Portugal; f School of Pharmacy and Biomolecular Sciences, University of Brighton, Brighton, United Kingdom; g Global Health and Tropical Medicine (GHTM), Instituto de Higiene e Medicina Tropical (IHMT), Universidade Nova de Lisboa, Lisbon, Portugal

## Abstract

*Mycobacterium avium* subsp. *paratuberculosis* is the causative agent of paratuberculosis. We report here the draft genome sequence of a rare pigmented *M. avium* subsp. *paratuberculosis* type C strain, comprising 58 contigs and having a genome size of 4,851,414 bp. The genome will assist in the execution of pigmentation and virulence studies on this mycobacterium.

## GENOME ANNOUNCEMENT

*Mycobacterium avium* subsp. *paratuberculosis* infects a wide range of animals, including humans, and is the causative agent of paratuberculosis, which affects ruminants, camelids, rabbits, and hares (1–3). *M. avium* subsp. *paratuberculosis* strains are classified into two major groups: types C and S. Type C strains are isolated from several hosts and are the predominant strain type infecting cattle, while type S strains have a host species preference for sheep and goats (4–6). The two types can be distinguished by their growth characteristics in culture medium (growth of type S strains is considerably slower) and genotyping assays. While yellow pigmentation in type S strains has been observed, to date only a single report of a putative pigmented type C strain has been published (7), but the strain is unavailable. Recently, a yellow pigmented strain was isolated from a goat fecal sample from Azores, Portugal. This strain was confirmed to be type C, based on its growth characteristics and single nucleotide polymorphism (SNP) analysis (8) results. Here, we announce the complete genome sequence of this rare pigmented type C Portuguese strain.

DNA was extracted from a single colony using a QIAamp DNA minikit (Qiagen). High-throughput sequence data were generated with Ion Torrent PGM (Thermo Fisher Scientific, Inc., USA), using a 400-bp sequencing kit and a 316 V1 chip. A total of 3,009,190 reads were produced, with a mean length of 299 bp. The genome assembly was created with MIRA (9) using the raw reads with preprocessing modules included in the software. The assembly contains 58 contigs, which encompass 4,851,414 bp. The largest contig obtained was 521,843 bp long and the *N*_50_ value was 188,875 bp, while the GC content was 69.3%.

Structural genome annotation was performed with Prokka (10), through Prodigal (11), yielding 4,666 protein-coding genes, 3 rRNAs, 58 tRNAs, and 1 transfer-messenger RNA. Functional annotation was executed with HMMER (12) against the eggNOG version 4.5 (13) database. The search space was reduced by selecting only hidden Markov models from actinobacteria. A total of 4,178 HMMER hits (90.8%) were detected, and 3,425 unique nonsupervised orthologous groups were found. The most common orthologs found in the genome were as follows: members of the PPE family, specific to mycobacteria and possibly playing a role in infection and virulence (14); *S*-adenosyl-l-methionine-dependent methyltransferase activity; cytochrome P450; acyl-CoA synthetase; synthase; transport proteins; and virulence factor mammalian cell entry family proteins.

A relatedness analysis was performed using 10,515 identified variants. The pigmented strain and 47 other *M. avium* subsp. *paratuberculosis* (15) strains were compared by mapping the reads to the K10 *M. avium* subsp. *paratuberculosis* reference genome (14) using BWA-MEM (16) and calling variation with GATK Haplotype Caller (17). This resulted in a dendrogram, built with SNPRelate (18), with three clearly differentiated groups for the type C, type S pigmented, and type S nonpigmented strains. The pigmented *M. avium* subsp. *paratuberculosis* isolate was unequivocally placed among the type C clade, confirming it to be a type C *M. avium* subsp. *paratuberculosis* strain. The first genome sequence of a pigmented type C *M. avium* subsp. *paratuberculosis* strain will assist future research focusing on pigmentation and virulence mechanisms of this bacterium.

### Accession number(s).

This whole-genome shotgun project has been deposited in GenBank under accession number MWPB00000000.

## References

[1] Motiwala AS, Strother M, Amonsin A, Byrum B, Naser SA, Stabel JR, Shulaw WP, Bannantine JP, Kapur V, Sreevatsan S 2003 Molecular epidemiology of *Mycobacterium avium* subsp. *paratuberculosis*: evidence for limited strain diversity, strain sharing, and identification of unique targets for diagnosis. J Clin Microbiol 41:2015–2026. doi:10.1128/JCM.41.5.2015-2026.2003. 12734243 PMC154725

[2] Timms VJ, Gehringer MM, Mitchell HM, Daskalopoulos G, Neilan BA 2011 How accurately can we detect *Mycobacterium avium* subsp. *paratuberculosis* infection? J Microbiol Methods 85:1–8. doi:10.1016/j.mimet.2011.01.026. 21281678

[3] Feller M, Huwiler K, Stephan R, Altpeter E, Shang A, Furrer H, Pfyffer GE, Jemmi T, Baumgartner A, Egger M 2007 *Mycobacterium avium* subspecies *paratuberculosis* and Crohn’s disease: a systematic review and meta-analysis. Lancet Infect Dis 7:607–613. doi:10.1016/S1473-3099(07)70211-6. 17714674

[4] Stevenson K 2010 Comparative differences between strains of *Mycobacterium avium* subsp. *paratuberculosis*, p 126–137. *In* Behr MA, Collins DM (ed), Paratuberculosis organism, disease, control. CAB International, Oxfordshire, UK.

[5] Salem M, Heydel C, El-Sayed A, Ahmed SA, Zschöck M, Baljer G 2013 *Mycobacterium avium* subspecies *paratuberculosis*: an insidious problem for the ruminant industry. Trop Anim Health Prod 45:351–366. doi:10.1007/s11250-012-0274-2. 23054804

[6] Stevenson K 2015 Genetic diversity of *Mycobacterium avium* subspecies *paratuberculosis* and the influence of strain type on infection and pathogenesis: a review. Vet Res 46:64. doi:10.1186/s13567-015-0203-2. 26092160 PMC4473831

[7] Watt J 1954 Johne’s disease in a bovine associated with the pigmented strain of *Mycobacterium johnei*. Vet Res 66:387.

[8] Leão C, Goldstone RJ, Bryant J, McLuckie J, Inácio J, Smith DGE, Stevenson K 2016 Novel single nucleotide polymorphism-based assay for genotyping *Mycobacterium avium* subsp. *paratuberculosis*. J Clin Microbiol 54:556–564. doi:10.1128/JCM.01958-15. 26677250 PMC4767949

[9] Chevreux B, Pfisterer T, Drescher B, Driesel AJ, Müller WEG, Wetter T, Suhai S 2004 Using the miraEST assembler for reliable and automated mRNA transcript assembly and SNP detection in sequenced ESTs. Genome Res 14:1147–1159. doi:10.1101/gr.1917404. 15140833 PMC419793

[10] Seemann T 2014 Prokka: rapid prokaryotic genome annotation. Bioinformatics 30:2068–2069. doi:10.1093/bioinformatics/btu153. 24642063

[11] Hyatt D, Chen GL, Locascio PF, Land ML, Larimer FW, Hauser LJ 2010 Prodigal: prokaryotic gene recognition and translation initiation site identification. BMC Bioinformatics 11:119. doi:10.1186/1471-2105-11-119. 20211023 PMC2848648

[12] Eddy SR 2011 Accelerated profile HMM searches. PLoS Comput Biol 7:e1002195. doi:10.1371/journal.pcbi.1002195. 22039361 PMC3197634

[13] Powell S, Forslund K, Szklarczyk D, Trachana K, Roth A, Huerta-Cepas J, Gabaldón T, Rattei T, Creevey C, Kuhn M, Jensen LJ, von Mering C, Bork P 2014 eggNOG v4.0: nested orthology inference across 3686 organisms. Nucleic Acids Res 42:D231–D239. doi:10.1093/nar/gkt1253. 24297252 PMC3964997

[14] Li L, Bannantine JP, Zhang Q, Amonsin A, May BJ, Alt D, Banerji N, Kanjilal S, Kapur V 2005 The complete genome sequence of *Mycobacterium avium* subspecies *paratuberculosis*. Proc Natl Acad Sci U S A 102:12344–12349. doi:10.1073/pnas.0505662102. 16116077 PMC1194940

[15] Bryant JM, Thibault VC, Smith DGE, McLuckie J, Heron I, Sevilla IA, Biet F, Harris SR, Maskell DJ, Bentley SD, Parkhill J, Stevenson K 2016 Phylogenomic exploration of the relationships between strains of *Mycobacterium avium* subspecies *paratuberculosis*. BMC Genomics 17:79. doi:10.1186/s12864-015-2234-5. 26813574 PMC4729121

[16] Li H 2013 Aligning sequence reads, clone sequences and assembly contigs with BWA-MEM. http://arxiv.org/abs/1303.3997.

[17] Van der Auwera GA, Carneiro MO, Hartl C, Poplin R, Del Angel G, Levy-Moonshine A, Jordan T, Shakir K, Roazen D, Thibault J, Banks E, Garimella KV, Altshuler D, Gabriel S, DePristo MA 2013 From FastQ data to high-confidence variant calls: the genome analysis toolkit best practices pipeline. Curr Protoc Bioinformatics 43:11.10.1–11.10.33. doi:10.1002/0471250953.bi1110s43 25431634 PMC4243306

[18] Zheng X, Levine D, Shen J, Gogarten SM, Laurie C, Weir BS 2012 A high-performance computing toolset for relatedness and principal component analysis of SNP data. Bioinformatics 28:3326–3328. doi:10.1093/bioinformatics/bts606. 23060615 PMC3519454

